# Oxidative Stress and Vascular Dysfunction in the Retina: Therapeutic Strategies

**DOI:** 10.3390/antiox9080761

**Published:** 2020-08-17

**Authors:** Yue Ruan, Subao Jiang, Aytan Musayeva, Adrian Gericke

**Affiliations:** Department of Ophthalmology, University Medical Center, Johannes Gutenberg University Mainz, 55131 Mainz, Germany; sjiang@uni-mainz.de (S.J.); ayten.musayeva@yahoo.com (A.M.)

**Keywords:** oxidative stress, reactive oxygen species, retinal disease, vascular endothelium

## Abstract

Many retinal diseases, such as diabetic retinopathy, glaucoma, and age-related macular (AMD) degeneration, are associated with elevated reactive oxygen species (ROS) levels. ROS are important intracellular signaling molecules that regulate numerous physiological actions, including vascular reactivity and neuron function. However, excessive ROS formation has been linked to vascular endothelial dysfunction, neuron degeneration, and inflammation in the retina. ROS can directly modify cellular molecules and impair their function. Moreover, ROS can stimulate the production of inflammatory cytokines, such as tumor necrosis factor-alpha (TNF-α) and interleukin-6 (IL-6) causing inflammation and cell death. However, there are various compounds with direct or indirect antioxidant activity that have been used to reduce ROS accumulation in animal models and humans. In this review, we report on the physiological and pathophysiological role of ROS in the retina with a special focus on the vascular system. Moreover, we present therapeutic approaches for individual retinal diseases targeting retinal signaling pathways involving ROS.

## 1. Introduction

Oxidative metabolism occurs primarily in the mitochondrion and the endoplasmic reticulum of eukaryotic living cells. In the mitochondrion, reactions of oxygen occur mainly in the electron transport chain (ETC) and often result in highly stable reaction products, such as adenosine triphosphate (ATP) and H_2_O. However, the ETC is not entirely efficient, so there is a basal level of electron leak under even the most optimal conditions. The inadvertent leakage of electrons and their reaction with molecular oxygen are major contributors to the production of cellular reactive oxygen species (ROS) [1,2]. Free radicals are atoms or molecules, which have one or more unpaired electrons in their outermost orbital shell, which makes them strongly reactive and thus capable of undergoing chain reactions responsible for the oxidative damage of cells and tissues [3].

Free radicals are classified into ROS, reactive nitrogen species (RNS), reactive iron species (RIS), and reactive copper species (RCS) [4,5,6]. It has been calculated that each cell is exposed to around 10,000–20,000 oxygen free radicals per day, whereas excess ROS as byproducts from metabolic processes can induce severe oxidative damage to lipids, proteins and nucleic acids, which may result in cell dysfunction and death, tissue damage and inflammatory responses, thus becoming a pivotal mechanism in numerous disease processes [1,7,8]. Oxidative damage is caused by a disturbance of the balance between the generation of ROS and the detoxification ability of the antioxidant defense systems, which include enzymes, such as superoxide dismutase (SOD), heme oxygenase, and catalase as well as direct antioxidants, such as glutathione, vitamin E, and vitamin C [9,10]. 

**Figure 1:**
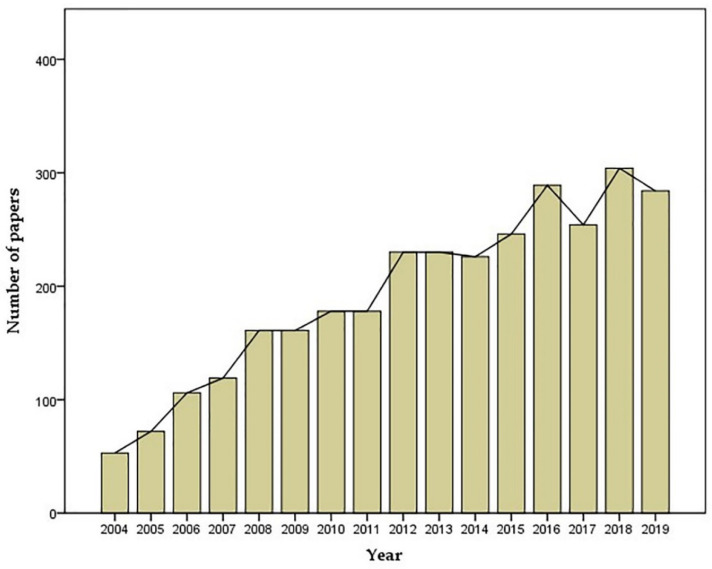
Yearly number of publications containing the search term “oxidative stress AND retina” from 2004–2019 [12].

ROS include radicals, such as superoxide (O_2_^•^^−^), hydroxyl (^•^OH), hydroperoxyl (HOO^•^), peroxyl (ROO^•^), alkoxyl (RO^•^), as well as nonradicals, such as hydrogen peroxide (H_2_O_2_), ozone (O_3_), singlet oxygen (^1^△g) and hypochlorous acid (HOCl) [11]. The term, oxidative stress, refers to the imbalance between the production of ROS and the ability of antioxidant defense systems to eliminate them, resulting in excessive ROS concentrations. Since elevated ROS levels have been linked to a variety of diseases, an increasing number of laboratories have been studying oxidative stress in many branches of medicine, including ophthalmology. From 2004 to 2019 more than 2733 publications on the topic of “oxidative stress and retina” have been published in Pubmed with a growing trend (Figure 1).

Retinal diseases, such as diabetic retinopathy (DR), glaucoma, age-related macular degeneration (AMD), and retinal vascular occlusion are frequent and vision-threatening. The increasing number of studies on retinal diseases led to a better understanding of their pathophysiology and yielded new therapeutic approaches [13,14,15,16]. The objective of this review is to summarize the current state of research on oxidative stress in the retina with a special focus on vascular function. Moreover, we discuss the role of ROS in the pathophysiology of retinal diseases and the therapeutic options.

## 2. Vascular Supply of the Retina

The retina is a light-sensitive layer that transmits visual information from the external world via the optic nerve to the visual center of the brain [17]. The neural retina (neuroretina) is composed of nine layers, the internal limiting membrane, nerve fiber layer, ganglion cell layer, inner plexiform layer, inner nuclear layer, outer plexiform layer, outer nuclear layer, external limiting membrane, and the rod and cone layer (photoreceptor layer). Light needs to traverse the retinal layers before initiating signal transduction in the rods and cones [17]. The output neurons of the retina are the ganglion cells. They are localized inside of the inner nuclear layer, which contains the cell bodies of bipolar, horizontal, and amacrine cells. The outer nuclear layer contains the rod and cone granules. Rods and cones are the photosensors responsible for phototransduction and are located outermost in the neural retina close to the pigment epithelium and the choroid. The photic input of the visual image is transmitted by the ganglion cell axons to the brain [18]. The retinal pigment epithelium (RPE) is composed of cells containing long sheet-like apical microvilli that interact with the tips of the cylindrical photoreceptor outer segments extending from the outer retinal surface. The basal surface of the RPE interacts with the Bruch’s membrane [19,20]. The RPE is essential for maintaining the homeostasis of the retina by directional transport of nutrients and removal of the shedding photo-oxidized photoreceptor outer segment membranes [20]. The RPE forms the outer blood-retinal barrier (oBRB) and is attached to the choroid. Histologically, the choroid is composed of five layers: the Bruch’s membrane, the choriocapillaris, the Sattler’s layer, the Haller’s layer, and the suprachoroid. The choriocapillaris, the Sattler’s layer, and Haller’s layer are vascular layers, which contribute to the rich choroidal circulation supplying the retinal photoreceptors and the retinal pigment epithelium with nutrients and oxygen [21].

**Figure 2:**
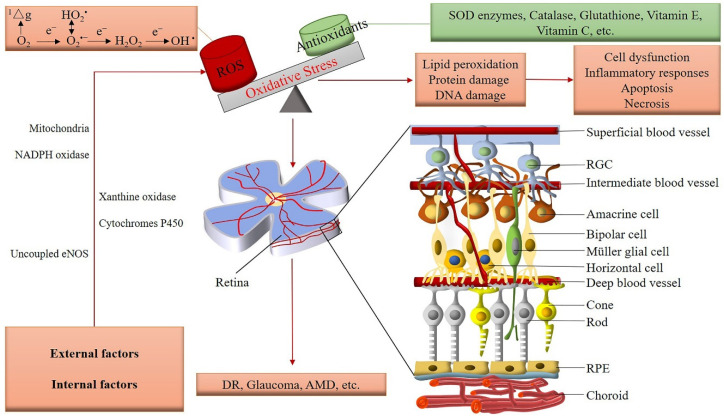
Oxidative stress in the retina (ROS: reactive oxygen species; ^1^△g: singlet oxygen; O_2_^•^^−^: superoxide; HO_2_^•^: hydroperoxyl; H_2_O_2_: hydrogen peroxide; ^•^OH: hydroxyl; NADPH oxidase: nicotinamide adenine dinucleotide phosphate oxidase; eNOS: endothelial nitric oxide synthase; SOD: superoxide dismutase; RGC: retinal ganglion cell; RPE: retinal pigment epithelium; DR: diabetic retinopathy; AMD: age-related macular degeneration).

The retina is supplied with blood, nutrients, and oxygen by two distinct vascular systems, the central retinal artery system (inner retina) and the choriocapillaris system (outer retina). The retina as one of the most metabolically active tissues requires high levels of oxygen and nutrients to maintain normal visual function [22,23]. Blood vessels emerging from the central retinal artery supply the inner half of the retina. The vessels branch into three capillary layers (superficial, intermediate, deep) after entering the eye through the center of the optic nerve. Retinal ganglion cells (RGCs) are neurons located near the inner surface of the retina. Apart from their essential role for the integration and transmission of visual information from photoreceptors to the brain, they provide angiogenic factors, such as hypoxia-inducible factor-1α (HIF-1α) during the development of the retinal vasculature [24,25]. The ganglion cell layer is vascularized by the intermediate and superficial vascular plexuses [26]. Retinal blood vessels, ganglion cells, and glial cells collectively form the neurovascular unit, a physical and functional unit, which participates in the regulation of retinal blood flow [27,28,29]. The retinal neovascular unit modulates vasodilation and vasoconstriction as the main basic regulatory mechanism of the retinal circulation and has a high metabolic demand, which makes it susceptible to damage by oxidative stress (Figure 2) [30].

## 3. Regulation of the Retinal Vasculature and Involvement of ROS

The brain and the retina are the highest energy-demanding systems in the body. The human retina utilizes around 50% more energy per gram than the human brain [31]. Its energy demands are met almost by oxidative metabolism and glycolytic metabolism and require nutrients and oxygen from the retinal vascular system [31,32]. The outer half of the retina, rich in oxidative enzymes and containing the photoreceptors, is supplied by the choroidal circulation with blood. Choroidal blood vessels are regulated by the sympathetic and parasympathetic nervous systems [31,33]. Although the retina is in high demand for oxidative metabolism, it needs to maintain transparency as a visually conductive tissue and therefore has a particularly low density of blood vessels [34]. Regulatory mechanisms of retinal blood flow for the inner retinal layers substantially differ from those of the choroidal circulation supplying the outer retinal layers. For example, retinal blood vessels have no autonomic innervation and a pronounced autoregulation [35,36,37,38]. The choroidal tissue is richly innervated by sympathetic, parasympathetic, and trigeminal sensory nerve fibers, which contribute to the regulation of the choroidal circulation [39]. Several studies reported that the choroidal circulation is not autoregulated [40,41,42]. However, other authors found some evidence for autoregulation in the choroidal vasculature [39,43].

Retinal circulation appears to be regulated not only by the blood vessels itself, but also by the surrounding tissue, the retinal neurovascular unit, a term first mentioned by Newman et al. [44]. The retinal neurons, glial cells, and the vascular endothelium form a complex physical and functional coupling unit, and each component of the unit works in close coordination to integrate retinal blood flow with metabolic activity [44,45].

The retinal vascular endothelium consists of a monolayer of thin squamous cells, which line the internal surface of retinal blood vessels. The endothelial cells form the inner blood-retinal barrier (iBRB) and play a major role in regulating vascular diameter and anti-inflammatory effects [36,46]. In addition to regulating retinal vascular tone, factors released by endothelial cells, such as nitric oxide (NO), inhibit both leukocyte adhesion and migration [47].

Nitric oxide (NO) is one of the most powerful vasodilatory factors. It is generated by the NO synthase (NOS) isozymes, endothelial NOS (eNOS), neuronal NOS (nNOS), and inducible NOS (iNOS), respectively. In the vascular endothelium, NO can be generated by eNOS and nNOS utilizing L-arginine as a substrate [38,48].

The NOS cofactor, tetrahydrobiopterin (BH4), is an essential activity regulator for all NOS isoforms. Loss or oxidation of BH4 to 7,8-dihydrobiopterin (BH2) is associated with NOS uncoupling, which results in the generation of superoxide rather than NO [49].

Pericytes play an important role in stabilizing the retinal vascular endothelium. They are generally separated from endothelial cells by the basal lamina, but also form peg-socket contacts with endothelial cells through holes in the basal lamina. Moreover, they help to maintain the blood-retinal barrier and contribute to maturation and stabilization of endothelial cells [50]. Previous studies found that through controlled platelet-derived growth factor (PDGF)-B/PDGF receptor beta (PDGFRβ) signaling, pericytes are essential for the formation of the blood-retinal barrier and respond to chemical messengers, such as angiopoietin-2 and vascular endothelial growth factor (VEGF). Moreover, they are essentially involved in blood flow regulation, especially in the capillaries [51]. RGCs are neurons located in the ganglion cell layer (GCL), the innermost cellular layer of the retina. Apart from their role of transmitting visual information from the retina to the brain, RGCs play a key role in retinal vascular development. RGCs are classified by Feng et al. into three subtypes based on their dendritic lamination pattern in the inner plexiform layer: On (direction-selective), Off (transient), and On-Off [52]. Since RGCs have a high metabolic demand, they are prone to become hypoxic. The ability of RGCs to sense hypoxia makes them crucial in the development of the normal retinal vasculature by regulating HIF-1α and VEGF-A expression [25]. VEGF-A is recognized as the principal regulator of retinal vascular growth. Sapieha et al. strongly supported the notion that the mechanism of VEGF-A generation may be orchestrated by the succinate receptor G protein-coupled receptor-91 (GPR91), which is expressed in response to increased succinate levels by RGCs [53]. Intriguingly, RGC-ablated retinas of adult rats did not show any proangiogenic effects [53,54]. The findings provide evidence that RGCs are key regulators in angiogenesis and play an important functional role in maintaining the normal structure and function of retinal blood vessels [55].

Glial cells have close contacts with both neurons and blood vessels. In addition, glia generates increases in intracellular Ca^2+^ in response to neuronal and vascular activity [56]. Retinal glial cells include Müller cells, astrocytes, and microglia. Müller cells release vasodilatory and vasoconstrictive metabolites, such as epoxyeicosatrienoic acids and 20-hydroxyeicosatetraenoic acid, in response to neuronal activity [56]. Müller cells also provide metabolic support for retinal neurons by regulating the transport and metabolism of neurotransmitters. The astrocytes play a critical role in the maintenance of the blood-retinal barrier (BRB) and have connections to retinal blood vessels and RGCs [30]. VEGF-A generated by astrocytes in the retina is an obvious component to integrate the close relationship between astrocytes and blood vessels [30]. Notably, the response of glial cells, including astrocytes, Müller cells, and microglia, to ROS is decisive for maintaining retinal health [57]. Müller cells, as the predominant retinal glial cells, play an essential role in the function and metabolism of retinal neurons by converting the neurotransmitter, glutamate, to glutamine [58]. Due to dysfunction of Müller cells in several retinal diseases, high glutamate concentrations are accumulating, which enhances the production of glutamate-induced ROS with toxic effects on retinal tissue [59]. Retinal astrocytes maintain the integrity of retinal neurovascular function, and their dysfunction contributes to the pathogenesis of various retinal pathologies, such as DR [60]. Under pathologic conditions, activated retinal microglia release proinflammatory cytokines and promote the production of ROS, which contribute to the apoptosis of retinal pericytes [61].

## 4. Pathophysiological Role of ROS in the Retinal Vasculature

ROS are produced in the body under physiological conditions, where they participate in normal cellular metabolism. The term, oxidative stress, refers to the imbalance between the production of ROS and the ability of antioxidant defense systems to eliminate them, resulting in excessive ROS concentrations. The excess of ROS becomes the primary cause of dysfunctional neurovascular units, which eventually leads to retinal vascular diseases [9,27,62]. Retinal endothelial dysfunction caused by ROS accumulation impairs the balance of NO metabolism and affects the responsiveness of vascular endothelial cells and smooth muscle cells to physiological stimuli, such as shear stress and vasoactive substances. The resulting endothelial dysfunction is characterized by reduced endothelium-dependent vasodilation and a proinflammatory and prothrombic state [46,63].

ROS generating processes are activated under various pathological conditions in the retina, such as DR, glaucoma, central retinal artery occlusion (CRAO), and AMD. These include enzymes, such as nicotinamide adenine dinucleotide phosphate (NADPH) oxidase, xanthine oxidoreductase, cytochrome P450, mitochondrial cytochrome oxidase, and uncoupled eNOS, which catalyze the excessive production of ROS in retinal tissue, including the vasculature [64,65]. Oxidation directly reduces BH4 bioavailability, but the oxidation products themselves, such as BH2, which have no cofactor activity, may compete with BH4 for binding to eNOS [49].

ROS can stimulate the production of inflammatory cytokines, such as tumor necrosis factor-alpha (TNF-α), interleukin- (IL-) 1-beta (IL-1β), and interleukin-6 (IL-6), and promote inflammatory processes by activating transcription factors, such as nuclear factor (NF)-kappa B (NF-κB) [66,67]. Moreover, ROS were demonstrated to induce activation of inflammasomes, which are multiprotein cytoplasmic complexes involved in mediating cellular inflammation [68]. One of the inflammasomes studied in depth is the NOD-like receptor family, pyrin domain-containing 3 (NLRP3) inflammasome, which induces inflammation by activation of caspase-1, followed by the cleavage and release of the proinflammatory cytokines, IL-1β and IL-18 [69]. In the retina, ROS were shown to induce an increase in retinal vascular permeability and retinal capillary cell apoptosis by increasing VEGF-A expression and activation of the NLRP3 inflammasome via the ROS-sensitive thioredoxin-interacting protein [70,71].

NADPH oxidases (NOX) are membrane-bound enzyme complexes, which largely contribute to ROS production by reducing oxygen molecules to ROS through NADPH-dependent single-electron reduction [72]. In humans, the NOX protein family contains 7 isoform-members, NOX1, NOX2, NOX3, NOX4, NOX5, dual oxidase 1 (DUOX1), and dual oxidase 2 (DUOX2) in various tissues and cells, of which NOX1 to NOX5 are six-transmembrane proteins and DUOX1 and DUOX2 seven-transmembrane proteins [73]. The transmembrane proteins transport electrons through membranes to generate superoxide from oxygen [74]. The isoforms, NOX1, NOX2, NOX4, and NOX5 were found in endothelial cells. In the retina, NOX1, NOX2, and NOX4 are the most well-studied isoforms [74,75,76,77,78]. NOX2, known as gp91phox, was the first NOX isoform to be identified and the most studied one in the research field of DR [79]. In addition, p40phox, p47phox, p67phox, small GTPase (Rac)1, and Rac2 are also important oxidase activators in supporting NOX activity and decreasing the bioavailability of NO [80]. Activated microglia induce the production of ROS in retinal microvascular cells by inducing NOX2 and NOX4 expression [61]. There is also evidence that NOX1, NOX4, and NOX5 mediate ROS production and thus enhance inflammation, vascular permeability, and neovascularization in various retinal diseases [77,81]. NOX5 is expressed in humans and in other species, such as cattle and rabbits, but is absent in the mouse and rat genome [82]. However, Deliyanti et al. studied vascular endothelial-cadherin^+^ NOX5^+^ transgenic mice expressing inducible human NOX5 in endothelial cells. The authors reported that retinal vascular permeability and the expression of inflammatory factors were increased in vascular endothelial-cadherin^+^ NOX5^+^ mice compared to wild-type mice and that the inhibition of NOX5 reduced damage to the retinal vasculature [77]. Other studies clearly demonstrate a major pathogenic role for NOX2 in promoting oxidative stress in DR and ischemia-reperfusion events [83,84,85]. For example, NOX2 was shown to be activated by GTPase and contributing to mitochondrial damage in DR [86]. Blockade of NOX4 by the NOX4 inhibitors, GKT136901 and GKT137831, significantly reduced ROS levels and cell death in retinal endothelium. Hence, NOX4 may be considered as a potential therapeutic target in patients with retinal vascular diseases [78].

Mitochondria are the primary source of ATP and ROS involved in metabolic processes. However, mitochondria-derived ROS (mtROS) play also a key role in the pathogenesis of retinal vascular diseases [87]. There is accumulating evidence showing that the overproduction of mtROS in retinal endothelial cells may induce apoptosis by p38 mitogen-activated protein kinase (p38 MAPK) through poly (ADP-ribose) polymerase (PARP) activation, in addition to increased production of VEGF, leading to retinal vascular endothelial dysfunction and retinal capillary cell apoptosis [88].

Retinal vascular eNOS is essential for maintaining proper vascular function by generating NO. In this process, BH4 plays a key role in maintaining eNOS homodimer stabilization and binding of L-arginine to the active site [89]. However, some pathological conditions can cause a decrease in BH4 levels with a consecutive dysfunction of eNOS, also known as eNOS uncoupling. Under such conditions, eNOS is “uncoupled” from the generation of NO and instead generates superoxide, which rapidly combines with NO to form the RNS, peroxynitrite (ONOO^−^). ONOO^−^ is a reactive oxidant, which in turn further contributes to a reduction of NO production and to endothelial dysfunction [90,91]. Based on in vitro findings in retinal endothelial cells, it has been suggested that oxidative injury caused by eNOS uncoupling may be reversed by supplementation of L-arginine or BH4 precursor [92].

## 5. ROS and DR

According to the World Health Organization, worldwide approximately 180 million people suffer from DR, one of the leading causes of visual impairment [93]. Bourne et al. reported that from 1990 to 2010 DR ranked as the fifth among other ocular diseases of treatable blindness [94]. DR is a metabolic disease whose underlying mechanisms have not been completely elucidated so far. However, experimental and clinical studies revealed that excessive ROS production plays a primary role in the pathogenesis of DR [95]. Persistent hyperglycemia stimulates the generation of ROS in the body, breaking the balance of the metabolic system. The overproduction of ROS induces the production of inflammatory mediators leading to cell injury, which forms a vicious circle both in retinal blood vessels and neurons, promoting the development of neuronal and vascular dysfunction [81]. A growing number of studies is recently reporting on the secondary effects of retinal neurons on retinal vascular tissue in DR, implying that DR is preferred to be considered as a retinal neurovascular disease rather than only a pure retinal vascular disease [96]. The changes caused by ROS in DR cannot be explained by only a single signaling pathway [97]. Major signaling pathways contributing to the pathophysiology of DR, in which ROS are involved, are the MAPK pathway, the polyol pathway, the renin-angiotensin system (RAS) pathway, the protein kinase C (PKC) pathway, the PARP pathway and the advanced glycation endproducts (AGEs) pathway [98,99,100,101].

Persistent hyperglycemia in DR activates the MAPK signaling pathway [100,102]. The MAPK signaling pathway includes three common members: p38, extracellular signal-related kinase (ERK), and c-Jun N-terminal kinase (JNK) [103]. JNK and p38 MAPK are known as stress-activated enzymes, which are triggered by hyperglycemia, ROS, or osmotic stress, whereas ERKs are activated through Ras-dependent signal transduction pathways by hormones and growth factors [104]. MAPK members are regulated by reversible phosphorylation of tyrosine and threonine residues, indicating that protein phosphatases play a critical role in regulating the activation status of these enzymes [105]. The MAPK pathway is also known to exert positive feedback mechanisms on ROS production in activation of the NF-κB pathway, AP-1, and p53 transcription factors [106,107]. Activation of these transcription factors can induce the expression of genes for a large number of inflammatory mediators [99]. Based on studies in rat models of DR, it has been suggested that the MAPK may be one of the chronic causes of inflammation and apoptosis in DR [107,108].

The increased intracellular glucose in a hyperglycemic environment leads to increased enzymatic conversion of glucose to the polyalcohol, sorbitol [107,109]. The polyol pathway is a two-step metabolic process, which is activated by hyperglycemia. In this process, aldose reductase (AR) reduces the excess glucose to sorbitol by using NADPH as a cofactor, causing an increase in oxidation of NADPH to NADP^+^ in vascular endothelial cells [110]. The reduction of NAD^+^ to NADH may trigger hypoxia and redox imbalance and increase intracellular NADH levels, which increases ROS and reduces the antioxidant capacity of retinal cells, such as retinal vascular endothelial cells, RGCs, Müller cells, and pericytes. ROS emerging from the polyol pathway are regarded to have multiple damaging effects in the retina [110,111]. It has also been suggested that Na^+^-K^+^-ATPase activity is decreased by increased flux through the polyol pathway and activation of PKC, causing an increase in cytosolic NADH/NAD^+^ and a decrease in cytosolic NADPH [107,112]. The increased sorbitol generated in the cell does not diffuse easily across cell membranes causing high sorbitol concentrations in vascular cells, which results in osmotic damage [112]. Conversely, Brownlee proposed that sorbitol concentrations measured in diabetic vessels and nerves are far too low to cause osmotic damage [107].

The renin-angiotensin system (RAS) is a hormonal system involved in blood pressure regulation. Intriguingly, a local RAS has been proposed for the eye [113]. Danser et al. measured the levels of Ang I and Ang II in the neural retina, retinal pigment epithelium, and choroid of porcine eyes, and found that Ang I and Ang II were 5- to 100-fold higher in these tissues than in the blood [114]. The local RAS is believed to be activated by hyperglycemia, which is reflected by elevated concentrations of prorenin and Ang II in the vitreous of diabetic patients [114,115]. It has been suggested that Ang II contributes to diabetic complications in the retina via activation of the angiotensin II receptor type 1 (AT_1_R) or the angiotensin II receptor type 2 (AT_2_R) in retinal blood vessels and neurons [107,116,117,118]. There is increasing evidence suggesting a crosstalk between the RAAS and ROS production in the retina [119]. For example, Ang II can activate NOX via the AT_1_R and, consequently, induce the generation of ROS in retinal blood vessels and Müller cells, eventually causing retinal vascular dysfunction and neurodegeneration [120]. Nagai et al. reported that the AT_1_R/NF-κB pathway contributes to hyperglycemia-induced inflammation in the retina [121].

PKC is a serine/threonine kinase, which is closely associated with the development of DR and plays an important role in numerous signaling pathways [122]. Diacylglycerol (DAG), a key second messenger in cells, is elevated in the hyperglycemic environment of the diabetic retina [123]. PKC isoforms activated by increased DAG levels also modulate neovascularization and vascular endothelial permeability by activating VEGF-dependent intracellular signal transduction pathways [124]. The PKC pathway is activated by ROS and in turn exacerbates the generation of ROS production, reduces the bioavailability of NO, increases endothelial permeability, and elevates VEGF expression in DR [107].

**Figure 3:**
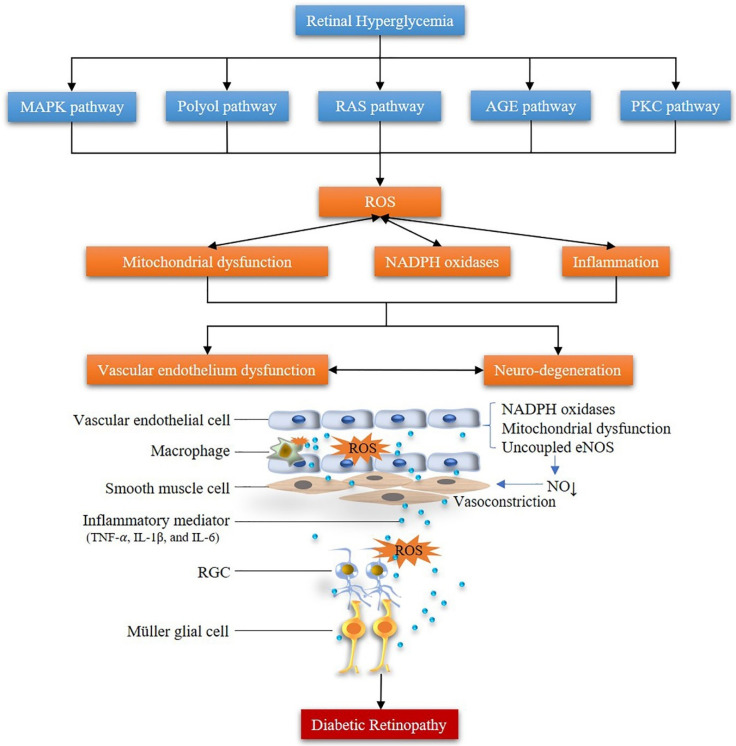
Pathophysiological mechanisms of ROS in diabetic retinopathy (MAPK: mitogen-activated protein kinase; RAS: renin-angiotensin system; PKC: protein kinase C; AGE: advanced glycation endproduct; ROS: reactive oxygen species; NADPH oxidase: nicotinamide adenine dinucleotide phosphate oxidase; eNOS: endothelial nitric oxide synthase; RGC: retinal ganglion cell).

High glucose levels promote the generation of AGEs, which in turn activate receptors for advanced glycation end products (RAGE) in retinal cells. Activation of RAGE triggers a range of pathogenic mechanisms contributing to DR [125]. For example, AGEs exert toxic effects in retinal pericytes by inducing ROS production and consequently apoptosis [126]. Sato et al. suggested that an interaction between toxic AGEs (TAGE) with RAGE mediates the release of pro-inflammatory molecules and promotes ROS generation, thus contributing to diabetic complications [127]. TAGE have been demonstrated to play a crucial role in the development of inflammatory reactions and microvascular disease in DR. Furthermore, TAGE were found to induce monocyte chemoattractant protein-1 (MCP1) in retinal vascular endothelial cells through intracellular ROS generation and VEGF expression (Figure 3) [127,128].

It has been suggested that in diabetic retinopathy, free fatty acids may induce oxidative stress, accelerate cell senescence, and promote the release of inflammatory cytokines, which are stimulators of leukostasis, vascular permeability, and basement membrane thickening [129,130]. Although the results of many human studies investigating the associations between DR and lipid abnormalities are inconsistent, there are hints, especially from cell culture and animal experiments that dietary modulation of specific lipids may help to prevent or to treat DR [131,132].

## 6. ROS and Retinal/Ocular Ischemia

Cessation of retinal blood flow as observed in CRAO is known to have a deleterious impact on visual acuity after a short period of time and presents an ophthalmic emergency, because of the risk of permanent vision loss and other ocular complications, such as retinal or vitreous hemorrhage, retinal neovascularization or neovascular glaucoma [133,134,135,136,137]. The ocular ischemic syndrome is typically less acute than CRAO and characterized by a reduced blood supply to both the retinal and choroidal circulation, which is mostly due to a stenosis of the common or internal carotid artery. The syndrome occurs mostly in elderly people, especially in men over 50 years [136]. Systemic and local arterial fibrinolysis have failed to improve the outcome of CRAO compared to conservative treatment (e.g., acetylsalicylic acid, ocular massage) or were even reported to be harmful [138,139]. Hence, there is a need for new therapeutic approaches aimed at increasing the tolerance of retinal tissue to ischemia.

At present, many experimental ischemic models have been established to discover the possible pathway and the therapy targets involved in retinal ischemia. Retinal damage following retinal ischemic injury involves the activation of NOX2 [85]. In a model of ischemia/reperfusion (I/R) in pigs with an ischemic period of 12 min followed by 20 h of reperfusion, retinal arterioles developed endothelial dysfunction and displayed increased levels of HIF-1α, VEGF-A, NOX2 and ROS [140].

Arginase as an important enzyme is known for its role in ammonia detoxification in the urea cycle of the liver and has also been found in the neurovascular system [141]. Arginase has two isoforms: arginase 1 (A1) and arginase 2 (A2) [142]. A1 is a cytosolic isoform abundantly expressed in the liver, but has also been detected in other tissues and blood cells [143,144,145]. A1 has been associated with endothelial dysfunction in various pathophysiological conditions, such as diabetes, and ischemia [146,147,148]. In contrast, A2 is a mitochondrial ubiquitous enzyme [149]. Both arginase isoforms are expressed in the retina [150]. Since arginase can compete with NOS for the common substrate, arginine, arginase can affect eNOS function and contribute to ROS generation in the pathogenesis of retinal ischemia [149]. Studies showed that A2 plays a major role in causing both vascular dysfunction and neuronal degeneration following retinal I/R injury by increasing ROS in the retina [149]. Deletion of A2 in mice reduced ROS levels, vascular dysfunction and RGCs death in I/R retinal injury [151].

Recent studies have shown that toll-like receptor 2 (TLR2) and TLR4 are involved in ischemic retinal injury [152]. It has also been shown that TLRs are activated by specific accessory proteins, such as myeloid differentiation protein 2 (MD2) and induce deleterious downstream effects in ischemic retinal injury [153]. The same study also reported that MD2 plays a key role in the pathogenesis of retinal I/R damage through the TLR4-NOX4 pathway and by ROS and inflammatory cytokine involvement [153]. Ulbrich et al. identified that the TLR2/TLR4/STAT3/NF-κB pathway is involved in generating ROS in a model of ischemia-reperfusion injury in the rat retina [152].

## 7. ROS and Glaucoma

Glaucoma is a serious ophthalmic disease characterized by progressive RGC and visual field loss and the second leading cause of irreversible blindness worldwide. The disease affects more than 60 million people over the world, and 8.4 million people of 60 million people were blind by 2010. Moreover, 11.1 million people were estimated to lose vision from primary glaucoma by 2020 [154]. Hence, glaucoma is considered a significant psychological and economic burden to individual patients and the society [155].

Although the mechanisms of visual loss are not fully understood in glaucoma, ROS play a major role in its pathophysiology [156,157]. In support of this concept, levels of ROS were shown to be increased in glaucoma patients both in aqueous humor and in blood serum [158]. One of the main risk factors for glaucoma is elevated intraocular pressure (IOP). Intriguingly, moderately elevated IOP was shown to induce excessive ROS levels, increased NOX2 expression, and endothelial dysfunction in retinal arterioles of mice, suggesting that IOP elevation compromises vascular function in the retina [159]. However, there are also other pathogenic mechanisms related to glaucoma, such as inflammation activated by ROS and glutamate excitotoxicity [160], which are not necessarily related to elevated IOP levels [156]. Tezel et al. presented evidence that RGC death during glaucomatous injury increased ROS generation in vitro [161]. Moreover, it has been shown that ROS regulate the immune response by stimulating the antigen-presenting ability of glial cells [161]. In the glaucomatous environment, glial cells produce ROS that damage the retina, and the elevated IOP-induces dysfunction of supportive glia, which may also facilitate secondary degeneration of RGCs in glaucoma [162]. Considering the interaction between the retinal vasculature and retinal neurons, the retinal neurovascular unit may play an important role in the pathogenesis of glaucoma and may become the therapeutic target in glaucoma [163,164].

AGEs in glaucomatous tissues have been detected in the axons of RGCs and retinal glial cells, which might have additional detrimental effects in the progression of vascular dysfunction and neurodegeneration [165]. The studies of AGEs are primarily focused on the mechanisms involved in diabetes and diabetes-related complications. However, substantial amounts of AGEs and RAGE have been found in optic nerve and retina, such as RGCs, glial cells, and Müller cells, in glaucoma, suggesting that the AGE pathway contributes to the pathogenesis of glaucoma, too [161,166]. The upregulated RAGE on RGCs and glial cells make them susceptible to AGE-mediated events through specific receptor-mediated signaling by activation of signaling molecules, such as MAPK and NF-κB, leading to ROS generation, immune responses, angiogenesis, and neuronal apoptosis in the glaucomatous retina [167]. A recent meta-analysis reported that hyperlipidemia was associated with an increased risk for glaucoma [168]. However, the original studies included in the meta-analysis displayed highly heterogenic results [168].

Also, the results of studies on the association of apolipoprotein E gene (ApoE) polymorphisms with glaucoma were heterogenic [169,170,171,172,173,174]. ApoE is a 36-kDa glycoprotein and a component of lipoprotein particles (LPPs) that include low-density lipoproteins, high-density lipoproteins, and chylomicron remnants [175]. Mutations of the ApoE gene are associated with hypercholesterolemia [176,177]. Interestingly, a study in mice found that ApoE deficiency was even protective against RGC death induced by elevated intraocular pressure or optic nerve crush [178]. In contrast, a study in a rat model of elevated IOP suggested that hypercholesterolemia is a risk factor for the development of glaucoma by increasing iNOS expression and oxidative stress [179].

Zadeh et al. detected endothelial dysfunction together with an increased vascular expression of the lectin-like oxidized low-density lipoprotein receptor-1 (LOX-1), a receptor for oxidized low-density lipoproteins (ox-LDL), in ApoE deficient mice [180]. Moreover, NOX2 expression as well as ROS levels were elevated in the vascular wall of retinal blood vessels from ApoE deficient mice suggesting a mechanism involving LOX-1, NOX2, and ROS in mediating retinal vascular endothelial dysfunction [180]. Although the retinal vasculature supplies the inner retinal layers, including the RGC layer, the mice displayed a normal RGC number, suggesting that redundant mechanisms exist in the inner retina to compensate for the lack of ApoE and for endothelial dysfunction [180]. Also, in a rabbit model of hypercholesterolemia, autoregulation of optic nerve head blood flow was impaired supporting the concept that hypercholesterolemia affects ocular vascular responses. However, functional and morphological alterations were also found in the retinas, including a reduction in RGC density, suggesting that hypercholesterolemia triggers glaucoma development [181].

Taken together, there are several pathways involved in the development of glaucoma, in which ROS seem to play an important role.

## 8. AMD

AMD is the leading cause of irreversible blindness in the elderly population of the developed countries [182]. There were approximately 170 million individuals affected by AMD worldwide in 2016, which is projected to increase to 288 million in 2040 [182,183]. In Germany, the global costs for AMD are 91.4 million € per year, while the yearly costs for AMD in the UK are 101.1 million € and in Italy 60.5 million € [182,184]. The most important risk factor for AMD is aging [184]. As the global aging population is increasing every year, this expenditure and the prevalence of AMD are expected to increase proportionally in the future [182]. Therefore, it is not surprising that AMD has become one of the most important ocular diseases in the elderly population.

AMD is a neurodegenerative disease that mainly affects the macular region in the retina, causing impairment of central vision, and deprives the ability of individuals to participate in basic activities in daily life [182]. Some studies hypothesize that the pathogenesis of AMD starts in the RPE, which is between the Bruch’s membrane and the neuroretina [185]. Although the etiology of AMD is still not exactly understood, AMD is divided into early and late stages. The early stage of AMD is typified by accumulating drusen deposits between Bruch’s membrane and the RPE, while the late stage is categorized into atrophic (dry) form and choroidal neovascularization (wet) form [186]. In wet AMD, many new pathological blood vessels from the choroid grow into the normally avascular sub-RPE and sub-retinal regions [187]. These new blood vessels lack the function of normal vessels and often cause leakage into the retina and hemorrhage [188].

The RPE plays a key role in maintaining retinal metabolism as a retinal blood barrier, delivering growth factors, and blood-derived nutrients to the outer segments of photoreceptors [189]. Another important function of the RPE is autophagy, a process of lysosome-mediated degradation of unnecessary cellular molecules and organelles, and elimination of aged or damaged metabolites or cells from photoreceptor cells in the retina. For example, the whole RPE phagocytes nearly 30,000 photoreceptor outer segments every day [190,191]. Aging is the main factor leading to age-related physiological changes in the RPE [20]. The metabolic and autophagy function of RPE determines a high metabolic demand in the RPE [191]. RPE is a tissue, which is enriched with mitochondria, due to the high metabolic activity from the RPE [192]. As a result, the RPE is a major source of ROS in the retina in AMD, leading to structural changes and damage in the progression of AMD [192].

Since the macula lacks a neuroretina, the macular region is especially susceptible to excessive ROS concentrations generated by the RPE [193]. Under pathological conditions, the RPE significantly produces high levels of ROS by the mitochondrial ETC complexes in mitochondria, principally by complexes I (NADH complex) and III (cytochrome complex), and ROS can leak out of the mitochondrial membrane [194]. In addition, photo-oxidative stress is a major source of oxidative stress from processing light for vision [195]. Terluk et al. have demonstrated that mtDNA damage preferentially develops in the RPE, but not the retina, illustrating the significance of mtDNA damage, which leads to RPE dysfunction and death in the progression of AMD [196]. These changes are associated with decreased peroxisome proliferator-activated receptor gamma coactivator 1-alpha (PGC-1α), which is a regulator of mitochondrial biogenesis and function, and NAD-dependent deacetylase sirtuin1 (SIRT1) [185].

Studies in genetically modified animal models of atherosclerosis reported on pathological changes in outer retinal layers, such as lipoidal degeneration and basal deposits in the Bruch’s membrane, which were similar to changes observed in aging human eyes, with some functional and morphologic alterations resembling those found in AMD [197,198,199,200]. A study in ApoE deficient mice suggested that zeaxanthin, a non-provitamin A carotenoid that belongs to the xanthophyll family, and other antioxidants may reduce VEGF expression in RPE-choroid homogenates and delay or reverse alterations in the RPE and deposits in the Bruch’s membrane [201]. Similarly, in a porcine model of hypercholesterolemia, ultrastructural alterations, such as pyknosis, irregular nuclear membranes, and cytoplasmic accumulation of lipids and autophagocytic vacuoles were observed in RPE cells [202].

In support of this concept, some studies in humans found a positive association of cholesterol intake with AMD [203,204]. Moreover, some epidemiologic studies reported a link between increased HDL cholesterol levels and AMD [205,206]. Genome-wide association studies also identified several HDL cholesterol genes associated with AMD susceptibility [207,208,209,210].

## 9. Therapeutic Strategies to Reduce Oxidative Stress

Recent studies have shown that prevention of ROS formation significantly reduces retinal damage associated with retinal vascular dysfunction and neurodegeneration in retinal diseases, including DR, hypercholesterolemia, retinal ischemia, glaucoma, and AMD. Thus, continued investigation of the benefits of ROS suppression in retinal diseases is warranted [211].

The current treatments have been suggested to target the complex activated signaling pathways of DR, which include glucocorticoid therapy (triamcinolone acetonide), which inhibits the NF-κB and MAPK pathways, RAAS blockers, and PKC inhibitors [212]. The intravitreal injection of triamcinolone acetonide (IVTA) has been part of clinical practice for over 50 years [213]. Zhang et al. showed that IVTA inhibits the p38 MAPK pathway, which exerts neural protective effects on retinal neurons in diabetes [212]. The AT_1_R blocker, candesartan, has been tested in clinical trials by Sjølie et al. and was shown to improve mild to moderate retinopathy in patients with type 2 diabetes [214]. Ruboxistaurin, a PKC beta inhibitor, has been demonstrated to ameliorate diabetic macular edema in clinical trials [215,216]. In diabetic retinopathy, free fatty acids may induce oxidative stress. The peroxisome proliferator-activated receptor-β/δ (PPARβ/δ) is a known target of lipid signaling, suggesting a potential role for this transcription factor in fatty acid-induced retinal inflammation. The PPARβ/δ antagonist, GSK0660, was shown to reduce retinal cell inflammation and leukostasis suggesting that inhibition of the PPARβ/δ pathway may be a therapeutic target for DR [130].

In a clinical study conducted in patients with primary hypercholesterolemia in 2013 at 198 sites in 17 countries, evolocumab, an inhibitor of proprotein convertase subtilisin/kexin type 9 (PCSK9), has been combined with moderate- or high-intensity statin therapy and was shown to additionally reduce LDL-C levels [217]. Moreover, in previous studies, curcumin has become a safe and potential treatment that can scavenge ROS and limits the risk of lipid peroxidation [218].

In 2014, the therapeutic effects of PPARα agonists in I/R injury have been studied and revealed that PPARα activation can alleviate oxidative stress by downregulating HIF-1α/NOX4 in the ischemic retina [219]. In addition, the study showed that octreotide, a somatostatin analog, had protective effects on I/R-treated retinal tissue by mitigating oxidative stress and downregulating NF-κB and intercellular adhesion molecule-1 expression [220]. Moreover, water-dispersible hesperetin was shown to protect RGCs by decreasing I/R-induced ROS [221]. Furthermore, Wang et al. have reported that laminin α4 (LAMA4) is involved in the development of glaucoma and that downregulation of LAMA4 might reduce ROS-induced apoptosis of RGCs by inhibiting the activation of the MAPK signaling pathway [222]. In another study, neurotrophins (NTs) were shown to be promising agents for neuroprotection activated by NT signaling through Trk receptors by the PI3-K-Akt-TOR and the MEK/ERK1/2/MAP kinase pathways, evoking protective and cell survival responses in the retina [223]. A small-molecule inhibitor (tozasertib) was shown to inhibit the JNK pathway and to promote RGC survival in an in vivo rodent optic nerve transection model [223,224].

Prokai reported that estrogens may contribute to the neuroprotection of neurons to oxidative stress [225]. 17β-Estradiol eye drops have been designed and studied to investigate the neuroprotection on RGCs in a model of glaucoma [226]. Memantine hydrochloride, an uncompetitive the *N*-methyl-D-aspartate antagonist, was originally used for Alzheimer’s disease and approved in Europe in 2002 for treatment of moderate-to-severe Alzheimer’s disease [227]. Experimental glaucoma studies in monkeys showed that daily oral dosing of 4 mg/kg memantine was both safe and effective in reducing the injury and functional changes from chronic ocular hypertension [228]. However, in a clinical phase 3 study, daily treatment with oral memantine over 48 months failed to provide neuroprotection in glaucoma in patients [229]. Osborne suggested that the death of RGCs in glaucoma was induced by different causes and one of the triggers could be the excessive activation of the *N*-methyl-D-aspartate receptors, suggesting that memantine could still be worth to explore as a potential neuroprotective strategy in glaucoma [230].

So far, there is still no compelling clinical treatment for dry AMD. Therefore, looking for effective prevention and therapeutic strategies needs to be continued. In the Age-Related Eye Disease Study (AREDS) in 2001, 3640 enrolled participants had a statistically significant reduction of the development of advanced AMD when receiving antioxidants (vitamin C, 500 mg; vitamin E, 400 IU; and β-carotene, 15 mg) plus zinc [231]. However, β-carotene is barely detectable in the human retina, and some clinical trials reported a potential risk for developing lung cancer in smokers with high-dose supplementation of β-carotene [232,233,234]. Lutein and zeaxanthin are found in the human retina, especially in the foveal region, since they are important components of the macular pigments [235]. The multicenter Eye Disease Case-Control study has shown in 1994 that lutein and zeaxanthin were most strongly related to a risk reduction for AMD [236]. Moreover, lutein and zeaxanthin have been studied extensively in various studies as important free radical scavengers in reducing the risk of progression from early AMD to late AMD [237]. The protective mechanisms of lutein could be considered to filter the blue light and to scavenge ROS induced by light [238]. Apart from the mechanisms above, a study revealed a differential mechanism for lutein in vascular diseases by reducing PDGF signaling, including phosphorylation of PDGFR-β and its downstream protein kinases/enzymes, such as MAPKs [239]. The AREDS2 was initiated to study the effects of the inclusion of lutein and zeaxanthin into the original AREDS formulation in 2006. The study reported that the AREDS2 formulation with the inclusion of lutein (10 mg/d) and zeaxanthin (2 mg/d) reduced the probability of advanced AMD in patients [240]. Nevertheless, more evidence for the effectiveness and safe dose range in lutein supplements is needed and remains to be established in further studies. Other therapies, such as the treatment with SUN N8075, a radical scavenger with neuroprotective properties, revealed protective effects on excess light-induced retinal damage by preventing ROS in the model of dry AMD in mice [241]. Edaravone, as a free radical scavenger, suppressing oxidative stress by scavenging ROS, has shown protective effects against retinal degeneration in experimental AMD models [242,243].

NADPH oxidases, as the masters of the ROS producing enzymes, became attractive therapeutic targets in various retinal diseases. NOX2 and NOX4 are considered to be the predominant catalytic subunits in the retina largely contributing to ROS generation, vascular endothelial dysfunction, and inflammatory cytokine expression [211]. The NOX4 inhibitors, GKT136901 and GKT137831, developed by GenKyoTex, are the best characterized NOX4 inhibitors currently available [244]. In experimental models of retinal vasculopathy by Appukuttan et al., GKT136901 and GKT137831 significantly reduced ROS generation and VEGF-A expression in retinal endothelial cells by inhibiting dimethyloxalylglycine (DMOG), which supported the therapeutic strategies of NOX4 inhibitors (GKT136901 and GKT137831) in retinal vascular diseases [78]. In a rat model of ischemic retinopathy, GKT137831 was shown to reduce inflammation by downregulating the pro-inflammatory phenotype of microglia [245]. Studies suggested that NOX2 knockout (KO) mice had an impaired immune defense against pathogens, and so therefore, complete inhibition of NOX2 activity may not be acceptable in humans [246,247]. The NOX2ds-tat peptide, a selective NOX2 inhibitor, has a low bioavailability, which limits its potential therapeutic options in humans [247].

Resveratrol is a natural polyphenol found in the skin of grapes, blueberries, raspberries, and peanuts, exhibiting anti-oxidative, anti-inflammatory, anti-apoptotic, and anti-aging effects [248]. At present, there has been no report on detrimental effects of resveratrol in both experimental animal and human studies, making it an attractive candidate for therapeutic use [249]. Li et al. reported that resveratrol was effective to prevent ROS generation in DR via inhibition of the ROS/AMPK/Sirt1/PGC-1α pathway [250]. Luo et al. demonstrated that resveratrol can prevent RGC death by blocking the Bax-caspase-3-dependent apoptotic pathway in I/R, and Luna et al. revealed that resveratrol effectively decreased the production of intracellular ROS and inflammatory mediators, such as IL-1α and IL-6 in glaucoma [251,252]. Furthermore, resveratrol was reported to reduce ROS production in the RPE in AMD by inhibiting the (MAPK)/ERK1/2 cascade. Thus, the MAPK/ERK1/2 cascade might be a therapeutic target for the prevention of AMD [211]. Together, these studies support the use of resveratrol as a possible therapeutic compound for retinal diseases.

Curcumin is another natural compound, which is a therapeutic candidate for a variety of retinal diseases due to its antioxidant properties [253]. Ran et al. reported that curcumin reduced ROS injury in human RPE cells through the ROS/PI3K/AKT/mTOR signaling pathway [254]. Cai et al. reported that by inhibiting p38 MAPK and NF-κB activation, curcumolide, a unique sesquiterpenoid from curcuma, alleviated retinal vascular permeability and the levels of ROS and TNF-α [255].

Various nutraceutical antioxidants exhibit numerous protective effects in the retina by directly scavenging free radicals. However, chronically applied antioxidants, such as vitamin C may remain to show compelling beneficial effects [256,257]. Some nutraceutical antioxidants show neuroprotective effects via activation of the nuclear factor erythroid-derived 2-related factor 2 (Nrf2) transcription factor pathway [258]. In the antioxidative process, Nrf2 activates the expression of phase II detoxifying enzyme genes through antioxidant response elements (AREs) in neurodegenerative diseases [259,260]. The Nrf2/AREs antioxidant pathway becomes a potential antioxidative target in organosulfur compounds nutraceuticals, such as allicin and L-sulforaphane [258]. Allicin modulated Nrf2 activation and protected cultured human umbilical vein endothelial cells against lipopolysaccharide-induced vascular oxidative stress [260]. Given the protective effects of Nrf2 activation, it is reasonable to assume that Nrf2 activators may provide significant therapeutic benefit against ROS in retinal vascular and neuron diseases. The first Nrf2 activator, dimethyl fumarate (Tecfidera^®^), has been approved by the U.S. Food and Drug Administration for the treatment of relapsing-remitting multiple sclerosis (RRMS) [261]. Zyla et al. showed that Tecfidera^®^ is effective in preventing or delaying recurrences of optic neuritis in patients with optic neuritis during RRMS in a retrospective study [262]. Dimethyl fumarate represented an efficient defensive strategy by activating the Nrf2 pathway to prevent the ROS-induced injury in RPE cells [263]. Wan et al. suggested that trimetazidine has antioxidative and anti-inflammatory effects by activating the Nrf2/heme oxygenase-1 (HO-1) pathway, contributing to the therapeutic effects against IOP-induced retinal damage [264]. A clinical trial with Tecfidera^®^ aimed at evaluating the safety and efficacy of the drug in dry AMD with geographic atrophy has been initiated in 2020 [265]. Table 1 summarizes the therapeutic strategies and their use for particular retinal diseases.

## 10. Conclusions

The neurovascular unit in the retina has gained much attention in recent years. Numerous studies demonstrated that ROS play a crucial role in the pathophysiology of various retinal diseases, contributing to vascular endothelial dysfunction and retinal neuron degeneration. Studies addressing the physiology and anatomy of the neurovascular unit led to a better understanding of the pathophysiology of ROS in retinal diseases. Therefore, therapeutic strategies targeting ROS pathways are more and more studied in ophthalmology. The development of therapeutic approaches that address ROS-induced retinal injury has the potential to profoundly impact the treatment of retinal diseases.

## Figures and Tables

**Table 1:** Therapeutic strategies and their use for particular retinal diseases.

Drug	Target	Research Type	Administration	Disease	Year	Effect	References
IVTA	p38MAPK	Animal experiment (Rat)	Intravitreal injection	DR	2013	Exerted neural protective effects on retinal neurons	[212]
Candesartan	RAAS	Clinical trial	Oral	DR	2008	Less severe retinopathy by the end of the trial was observed in the candesartan group	[214]
Ruboxistaurin	PKC β	Clinical trial	Oral	DR/Diabetic macular edema	2009	Ameliorated visual acuity decrease	[215]
GSK0660	PPARβ/δ	Cell culture	Cell culture	DR	2020	PPARβ/δ inhibition mitigated inflammatory signaling events elicited by metabolic stimuli and inflammatory cytokines.	[130]
Evolocumab	PCSK9 LDL-C	Clinical trial	Subcutaneous injection	Hypercholes-terolemia	2014	Reduced LDL-C levels in patients receiving statin therapy	[217]
Fenofibric acid (Feno-FA)	PPARα	Animal experiment (Mouse)	Intraperitoneal injection	Retinal ischemia	2014	Feno-FA treatment and PPARα overexpression decreased ROS levels and protected cultured retinal cells from hypoxic cell death	[219]
Octreotide (OCT)	NF-*κ*B	Animal experiment (Mouse)	Subcutaneous injection	Retinal ischemia	2015	Inhibition of ROS and downregulation of NF-κB and intercellular adhesion molecule-1 (ICAM-1) expression	[220]
Water-dispersible hesperetin (WD-Hpt)	ERK, IL-1β	Animal experiment (Mouse)	Intraperitoneal injection	Retinal ischemia	2015	Decreased I/R-induced ROS formation	[221]
17β-estradiol	Estrogen receptor	Animal experiment (Rat)	Tropical eye drops	Glaucoma	2014	Demonstrated significant neuroprotective effect	[226]
Memantine	*N*-methyl-D-aspartate (NMDA) receptor	Animal experiment (Monkey)	Oral	Glaucoma	2004	Both safe and effective for reduction of functional changes associated with chronic ocular hypertension	[228]
Memantine	NMDA receptor	Clinical trial	Oral	Glaucoma	2018	Daily treatment with memantine 10 mg or 20 mg for 48 months did not significantly delay glaucomatous progression	[229]
AREDS formulation	Free radicals	Clinical trial	Oral	AMD	2001	Demonstrated a statistically significant odds reduction for the progression of advanced AMD	[231]
Lutein/ zeaxanthin	Free radicals	Clinical trial	Oral	AMD	1994	Most strongly associated with a reduced risk for AMD	[236]
Lutein/ zeaxanthin	PDGF	Cell culture	Cell culture	Vascular disease	2012	Lutein could inhibit PDGFR signaling, whereas zeaxanthin did not	[239]
AREDS2 formulation	Free radicals	Clinical trial	Oral	AMD	2015	Lutein and zeaxanthin could be more appropriate than beta-carotene in the AREDS type supplements	[240]
SUN N8075	Free radicals	Animal experiment (Mouse)	Intraperitoneal administration	AMD	2014	Had protective effects on excess light-induced photoreceptor degeneration	[241]
Edaravone	Free radicals	Animal experiment (Mouse and common marmoset)	Intraperitoneal/ Intravenous injection	AMD	2016	Effective against laser-induced choroidal neovascularization (CNV) both in mice and in common marmosets	[242]
Edaravone	MAPKs	Animal experiment (Mouse)	Intraperitoneal/ Intravenous injection	AMD	2010	Significantly protected against light-induced photoreceptor degeneration at 5 days after exposure to light	[243]
GKT136901/ GKT137831	NOX1/NOX4 inhibitors	Cell culture	Cell culture	Retinal vasculopathy	2018	Treatment with GKT136901 or GKT137831 significantly reduced ROS production and VEGFA expression by endothelial cells	[78]
GKT137831	NOX1/NOX4 inhibitors	Animal experiment (Rat)	Subcutaneous injection	Retinal ischemia	2015	Reduced the increased leukocyte adherence to the vasculature and the pro-inflammatory phenotype of retinal immune cells	[245]
Resveratrol	AMPK /Sirt1/PGC-1α	Cell culture	Cell culture	DR	2017	Prevented ROS-induced apoptosis in high glucose-treated retinal capillary endothelial cells	[250]
Resveratrol	Bax-caspase-3	Animal experiment (Rat)	Intraperitoneal injection	Glaucoma/ Retinal ischemia	2018	Significantly reduced retinal damage and RGC loss	[251]
Curcumin	PI3K/AKT/ mTOR	Cell culture	Cell culture	DR	2018	Increased anti-inflammatory effect to high glucose-induced inflammatory injury in human retinal pigment epithelial cells	[254]
Dimethyl fumarate	Nrf2	Cell culture	Cell culture	AMD	2020	An efficient defensive strategy to prevent ROS-induced damage	[263]
Trimetazidine	Nrf2/HO-1	Animal experiment (Mouse)	Intraperitoneal injection	Glaucoma/ Retinal ischemia	2017	Attenuated retinal damage and RGC death, with a decrease in ROS and inflammatory cytokine production	[264]
Tecfidera^®^	Nrt2	Clinical trial	Oral	AMD	2020	Not finished yet	[265]

## Abbreviations

ACE	Angiotensin-converting enzyme
AGEs	Advanced glycation endproducts
AMD	Age-related macular degeneration
Ang	Angiotensin
ApoE	Apolipoprotein E
AREDS	Age-Related Eye Disease Study
AR	Aldose reductase
AREs	Antioxidant response elements
AT_1_R	Angiotensin II receptor type 1
AT_2_R	Angiotensin II receptor type 2
A1	Arginase 1
A2	Arginase 2
ATP	Adenosine triphosphate
BH4	Tetrahydrobiopterin
BH2	7,8-dihydrobiopterin
BRB	Blood-retinal barrier
CVD	Cardiovascular disease
CRAO	Central retinal artery occlusion
DAG	Diacylglycerol
DMOG	Dimethyloxalylglycine
DR	Diabetic retinopath
DUOX1	Dual oxidase 1
DUOX2	Dual oxidase 2
ETC	Electron transport chain
eNOS	endothelial nitric oxide synthase
ERK	Extracellular signal-related kinase
FA	Fenofibric acid
FH	Familial hypercholesterolemia
GCL	Ganglion cell layer
GPR91	Succinate receptor G protein-coupled receptor-91
HO-1	Heme oxygenase-1
H_2_O_2_	Hydrogen peroxide
HO_2_^•^	Hydroperoxyl
HOC1	Hypochlorous acid
HIF-1α	Hypoxia-inducible factor-1α
ICAM-1	Intercellular adhesion molecule-1
IL-1α	Interleukin-1 alpha
IL-β	Interleukin-1 beta
IL-6	Interleukin-6
IVTA	Intravitreal injection of triamcinolone acetonide
I/R	Ischemia/reperfusion
iBRB	Inner blood-retinal barrier
iNOS	inducible nitric oxide synthase
JNK	c-Jun N-terminal kinase
KO	Knockout
LDL-C	Low-density lipoprotein cholesterol
L	Lutein
mtROS	mitochondria-derived ROS
mtDNA	mitochondrial DNA
MCP1	Monocyte chemoattractant protein-1
MD2	Myeloid differentiation protein 2
NADPH oxidase	Nicotinamide adenine dinucleotide phosphate oxidase
NF-κB	nuclear factor-kappa B
NLRP3	Pyrin domain-containing 3
NMDA	N-methyl-D-aspartate
NOX	NADPH oxidases
NO	Nitric oxide
NOS	nitric oxide synthase
nNOS	neuronal nitric oxide synthase
NRF2	Nuclear factor erythroid-derived 2-related factor 2
NTs	Neurotrophins
O_2_^•−^	Superoxide
O_3_	Ozone
oBRB	Outer blood-retinal barrier
OCT	Octreotide
^•^OH	Hydroxyl radicals
ox-LDL	oxidized-LDL
ONOO^—^	Peroxynitrite
PARP	Poly (ADP-ribose) polymerase
PCSK9	Proprotein convertase subtilisin/kexin type 9
PDGF	Platelet-derived growth factor
PDGFRβ	Platelet-derived growth factor receptor beta
PKC	Protein kinase C
PPAR	Peroxisome proliferator-activated receptor
RAAS	Renin-angiotensin-aldosterone system
RAGE	Receptors for advanced glycation endproducts
RAS	Renin-angiotensin system
RCS	Reactive copper species
RGC	Retinal ganglion cell
RIS	Reactive iron species
RNS	Reactive nitrogen species
ROS	Reactive oxygen species
RPE	Retinal pigment epithelium
ROO^•^	Peroxyl
RO^•^	Alkoxyl
RRMS	Relapsing-remitting multiple sclerosis
SIRT1	NAD-dependent deacetylase sirtuin1
SOD	Superoxide dismutase
TAGE	toxic AGEs
TLR4	Toll-like receptor 4
TLR2	toll-like receptor 2
TNF-α	Tumor necrosis factor-alpha
VEGF	vascular endothelial growth factor
Z	Zeaxanthin
^1^△g	Singlet oxygen
